# M-CSF secreted by gastric cancer cells exacerbates the progression of gastric cancer by increasing the expression of SHP2 in tumor-associated macrophages

**DOI:** 10.18632/aging.205390

**Published:** 2023-12-29

**Authors:** Shaohua Zhang, Dongfei Ren, Huiyu Hou, Li Yao, Hufang Yuan

**Affiliations:** 1 Eighth People’s Hospital of Hebei Province, Shijiazhuang 050000, China; 2 HeBei General Hospital, Shijiazhuang 050000, China; 3 Handan Central Hospital, Handan 056000, China; 4 The Fourth Hospital of Hebei Medical University, Shijiazhuang 050000, China

**Keywords:** gastric cancer, tumor-associated macrophages, SHP2, GM-CSF, M-CSF

## Abstract

Objective: To investigate the effect of Src homology 2 domain-containing tyrosine phosphatase-2 (SHP2) in tumor-associated macrophages (TAMs), which is mediated by macrophage colony-stimulating factor (M-CSF) secreted by gastric cancer cells, on the development of gastric cancer and its molecular mechanism.

Methods: The progression of gastric cancer was detected by nude mouse tumor-bearing experiments. Colony formation assay and cell counting kit-8 (CCK8) assay were used to detect the proliferation capacity of gastric cancer cells. The migration capacity of gastric cancer cells was examined by wound healing assay. Transwell migration and invasion assays were performed on gastric cancer cells. Detection of relevant protein expression using western blotting.

Results: Overexpression of SHP2 could promote the progression of gastric cancer in nude mice. The results of colony formation assay and CCK8 assay showed that overexpression of SHP2 could enhance the proliferation of gastric cancer cells. It was found by wound healing assay and Transwell assay that overexpression of SHP2 could facilitate the migration and invasion of gastric cancer cells. The results of Western blotting revealed that overexpression of SHP2 could increase the expressions of p-STAT3, s-PD-1, p-Src, p-Lyn, p-PI3K, p-AKT, Arginase-1, MMP1 and MMP3 but decrease the expressions of TBK1 and SOCS1 in TAMs, and also increase the expressions of CD9, TSG101 and s-PD-1 in exosomes.

Conclusion: M-CSF secreted by gastric cancer cells can promote the proliferation, invasion and migration of gastric cancer cells by increasing the expression of SHP2 in TAMs.

## INTRODUCTION

Gastric cancer (GC) is a multifactorial disease influenced by both environment and heredity [1]. According to current statistics, GC is the fourth leading cause of cancer death worldwide, with a median survival rate of less than 12 months in advanced stages [2]. GC is a highly invasive malignancy with heterogeneity and remains a major global health concern [3]. Early diagnosis can improve the treatment success rate of GC [4]. Only 10% of people under the age of 45 are affected by GC, making it a rare disease [5, 6].

Immune cells in the tumor microenvironment play a key role in the progression of tumors. Tumor-associated macrophages (TAMs) are predominantly M2 phenotype [7]. In the tumor microenvironment, TAMs promote tumor progression through multiple mechanisms. First, TAMs can facilitate angiogenesis, increase tumor blood flow, and supply nutrients and oxygen to tumor cells [8]. Second, TAMs can enhance invasion and metastasis of tumor cells, and degrade extracellular matrix (ECM) by secreting matrix metalloproteinases (MMPs) and other protein-degrading enzymes, thus offering channels for tumor cells to penetrate the basement membrane [9]. Moreover, immunomodulation of macrophages has emerged as an attractive approach to anticancer therapy. Macrophage colony-stimulating factor (M-CSF) secreted by cancer cells binds to the colony-stimulating factor 1 receptor (CSF1R) on macrophages, which in turn activates downstream signaling pathways responsible for the M2 polarization of TAMs.

As a non-receptor protein tyrosine phosphatase, Src homology 2 domain-containing tyrosine phosphatase-2 (SHP2) is an important player in downstream cell signaling [10]. SHP2 is regulated by growth factors, cytokines and integrin receptors, and it is involved in cellular processes such as cell survival, proliferation and migration. SHP2 is critical in regulating biological responses to growth factors, hormones, cytokines and cell adhesion molecules, and is a key component of the signal transduction pathways that control tumor development and hematopoiesis [11]. Studies have shown that SHP2 can regulate a variety of cellular processes, including cell metabolism, growth, differentiation, migration, transcription, and oncogenic transformation [12]. Therefore, disorders of SHP2 expression or activity are associated with diseases. Moreover, overexpression or hyperactivation of SHP2 has been found in several solid tumors, including colorectal cancer, lung adenocarcinoma, glioblastoma multiforme, melanoma, neuroblastoma, liver cancer, prostate cancer and breast cancer [13, 14].

In this study, therefore, the effects of SHP2 in TAMs, which is mediated by M-CSF secreted by gastric cancer cells, on the proliferation, migration and invasion of gastric cancer cells and its molecular mechanism were investigated, which may provide an effective therapeutic target for GC.

## METHODS

### Cell culture

Human GC MGC-803 and BGC-823 cell lines as well as human monocyte THP-1 cells were purchased from Wuhan Procell Life Science and Technology Co., Ltd. MGC-803 and BGC-823 cells and THP-1 cells were cultured with RPMI-1640 medium containing 10% fetal bovine serum (FBS), 100 μg/mL streptomycin and 100 U/mL penicillin under the condition of 5% CO_2_, 37°C and saturated humidity. THP-1 cells were stimulated to differentiate into macrophages using M-CSF. The medium was changed every three days, and the cells in the logarithmic growth phase were collected for later experiments.

### Cell transfection and treatment

The SHP2 RNAi target NM_080549 NM_3 sequence was obtained from the database of the National Center for Biotechnology Information (NCBI), Bethesda, MD using online design software. The negative control (NC), si-SHP2 and SHP2-OE were not homologous to the target gene. THP-1 cells were transiently transfected using Lipofectamine 2000 (Invitrogen Life Technologies, Carlsbad, CA, USA) according to the manufacturer's instructions. Then they were co-cultured with MGC-803 and BGC-823 cells, respectively.

### Nude mouse tumor-bearing model

Six-week-old BALB/c-nu mice were purchased from Henan SCBS Biotechnology Co., Ltd. Each mouse was inoculated within 1 hour with the co-cultured MGC-803 and BGC-823 cells at a dose of 1 × 10^7^/200 μL. The needles were inserted into the upper waist of the nude mice, traveling to the head side, without piercing the skin or the muscle layer. The distance from the inoculated site was smaller than the length of the needle. Then the cells were temporarily stored in an ice box. After successful tumorigenesis, the tumor volume was routinely measured every other day in our laboratory.

### Colony formation assay

The GC cells in the logarithmic growth phase in each group were digested with 0.25% trypsin into single cells, and suspended in a complete medium for later use. The cell suspension was diluted in gradient, with three replicates in each group. Then 100 cells were inoculated in each dish containing 10 mL of 37°C pre-warmed culture medium, and evenly dispersed by gentle rotation, followed by culture in a 5% CO_2_ incubator at 37°C and saturated humidity for 2 weeks until visible colonies emerged in the dish. Then the supernatant was discarded, and the cells were carefully washed twice with PBS, and fixed with 5 mL of pure methanol or 1:3 acetic acid/methanol for 15 minutes. After the fixative was removed, the cells were stained with an appropriate amount of Giemsa staining solution for 30 minutes. Afterward, the staining solution was slowly washed away with running water, and the cells were air-dried. The plate was inverted and overlaid with a transparent film with grids. Then the colonies were directly counted with naked eyes or those with more than 10 cells were counted under a microscope. Finally, the colony formation rate was calculated.

### Cell counting kit-8 (CCK8) assay

MGC-803 and BGC-823 cells were seeded into 96-well plates and cultured in a 5% CO_2_ incubator at 37°C for 12 hours, with three replicates in each group, and PBS was added to the most marginal wells to reduce the impact of evaporation. Then 10 μL of CCK8 buffer was added directly into each well, and the cells were cultured in the 5% CO_2_ incubator at 37°C for another 2 hours. The optical density (OD) values at 450 nm were detected at 0 h, 24 h and 48 h with a microplate reader. Finally, the data were analyzed and the cell proliferation curves were plotted.

### Wound healing assay

First, some horizontal lines were drawn along a ruler using a marker pen at an interval of 0.5–1 cm on the back of a 6-well plate. The lines should cross the wells, and at least five lines were needed for each well. The cells in the logarithmic growth phase were trypsinized into single-cell suspensions and inoculated into the 6-well plate at 6 × 10^5^/well overnight. After the fusion rate reached 100% and the total volume of the final culture medium in each well was 2 mL, the cells were cultured at 37°C in a 5% CO_2_ incubator for 24 hours. The next day, the cells were washed three times with PBS using a 200 μL tip vertically with the plate. The floating cells from scratches were removed, and serum-free medium was added. Afterward, the images were acquired under a microscope (4×) at 0 and 48 h, and the wound distance was calculated using ImageJ software.

### Transwell assay

Invasion assay: Matrigel was diluted at 1:8, added to the upper chamber of the Transwell and incubated in an incubator at 37°C for 4 h to allow the Matrigel to gel. A single-cell suspension was prepared with serum-free medium and seeded into the Transwell chamber at an appropriate density. The Transwell chamber was then taken out, and the medium was discarded. The cells were washed twice with 1 × PBS, fixed with methanol for 30 minutes, and air-dried, followed by staining with 0.1% crystal violet for 15 minutes, and rinsing with 1 × PBS. The non-invading cells on the upper layer were gently wiped off with cotton swabs, and the invading cells were counted. Migration assay: No Matrigel was used, and the remaining operations were the same as those in the invasion assay. The migrating cells were counted directly under a microscope after staining.

### Isolation of exosomes

To generate exosome-free media, FBS was centrifuged overnight at 10,000 g and filtered through a 0.2 μm syringe filter (Millipore, Burlington, MA, USA). The exosome-free FBS was used for cell culture (DMEM supplemented with 10% exosome-free FBS). To isolate exosomes, cell supernatants were collected and centrifuged at 2000 g and 10,000 g at 4°C for 30 minutes, respectively. The final supernatant was filtered through a 0.22 μm syringe filter (Millipore Burlington, MA, USA) and centrifuged at 120,000 g for 70 minutes. Then spheroidal vesicles were washed with PBS and centrifuged again at 120,000 g for 70 minutes. The quality of exosomes isolated was verified by Western blotting of exosome markers and by nanoparticle tracking analysis and electron microscopy of their size and shape.

### Western blotting

THP-1 cells and isolated exosomes were lysed, and centrifuged to extract the proteins at 4°C. Then the proteins were loaded, subjected to SDS-PAGE, and transferred onto PVDF membranes. The membrane was blocked with 5% nonfat milk powder, and incubated with primary antibodies against SHP2 (Abcam, ab300579, 1:1000), TBK1 (Abcam, ab40676, 1:5000), SOCS1 (Abcam, ab280886, 1:1000), p-STAT3 (Abcam, ab76315, 1:2000), s-PD-1 (Abcam, ab214421, 1:1000), CD9 (Abcam, ab307089, 1:1000), TSG101 (Abcam, ab125011, 1:1000), p-Src (Abcam, ab185617, 1:5000), p-Lyn (Abcam, ab278639, 1:1000), p-PI3K (Abcam, ab182651, 1:500), p-AKT (Abcam, ab38449, 1:1000), Arginase-1 (Abcam, ab203490, 1:1000), MMP1 (Abcam, ab137332, 1:1000), MMP3 (Abcam, ab52915, 1:1000) and GAPDH (Abcam, ab8245, 1:10000). After washing three times with TBST, the membrane was incubated with secondary antibodies for 2 hours, and washed three times again with TBST. Finally, the protein expressions were analyzed.

### Statistical analysis

SPSS22.0 software was used for statistical analysis, and GraphPad Prism 9.0 was used for plotting. The results were expressed as mean ± standard error of mean. The comparisons were made between two groups by *t*-tests. The experiments were repeated at least three times in each group, and *P* < 0.05 was considered statistically significant.

## RESULTS

### SHP2 in TAMs could promote the progression of GC

**Figure 1:**
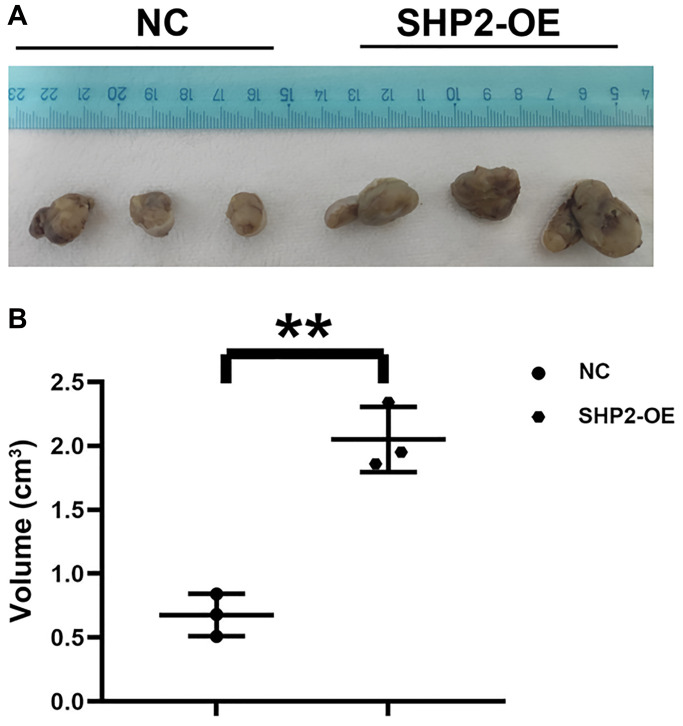
**SHP2 in TAM can promote the progression of gastric cancer.** (**A**) Results of nude mouse tumor-bearing experiment; (**B**) Tumor volume statistics of nude mice. ^**^P < 0.01.

The impact of SHP2 in TAMs on the development of GC was detected by nude mouse tumor-bearing experiments. The results showed that the tumor volume in the SHP2-OE group significantly increased compared with the NC group, suggesting that SHP2 in TAMs can promote the progression of GC (Figure 1).

### SHP2 in TAMs could enhance the proliferation of GC cells

**Figure 2:**
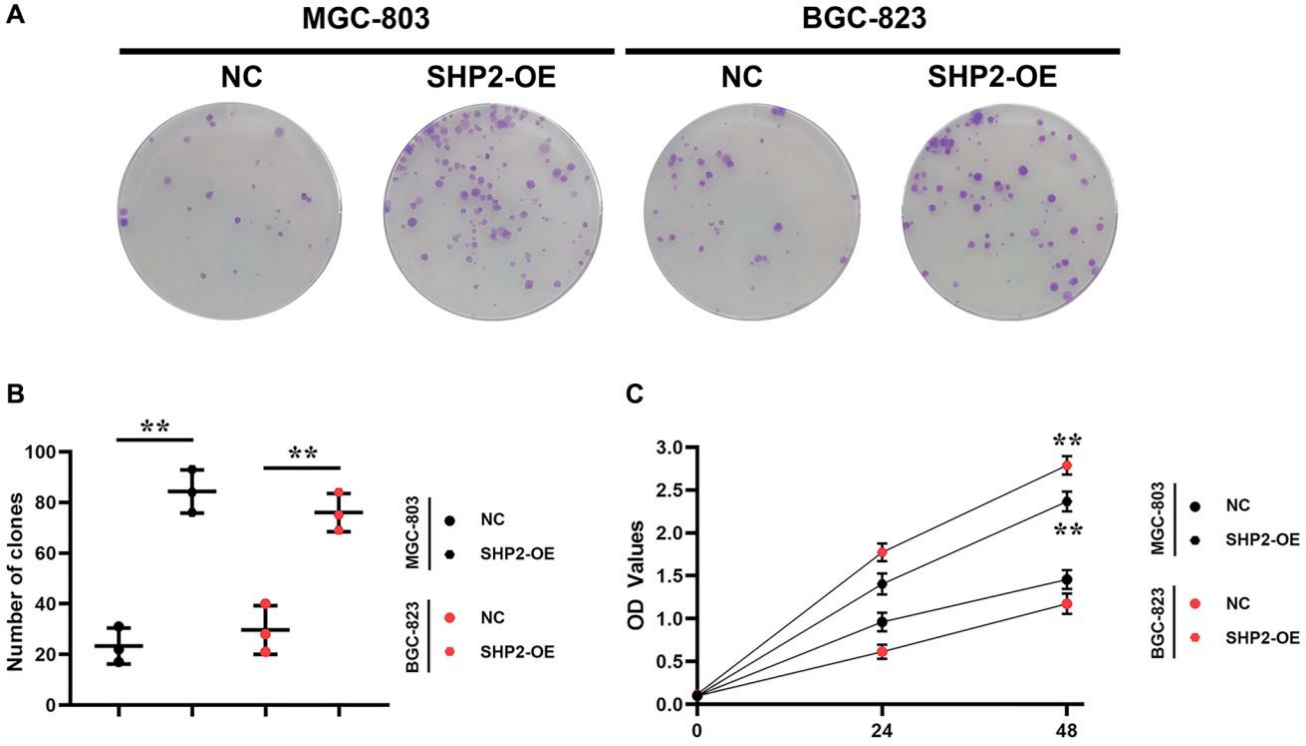
**SHP2 in TAM can promote the proliferation of gastric cancer.** (**A**) Results of colony formation assay; (**B**) Statistics of the clone formation cell number; (**C**) Results of CCK8 assay. ^**^P < 0.01.

Colony formation assay is one of the useful methods for assessing the cell proliferation capacity. Not every cell can proliferate to form colonies after adherence to the wall, but the cells that form colonies must have the ability to adhere to the wall and proliferate. The colony formation rate indicates the number of adherent cells surviving and forming colonies after inoculation, which can be used to reflect the cell proliferation capacity. The results of colony formation assay showed that overexpression of SHP2 could increase the number of colonies in MGC-803 and BGC-823 cells. Besides, the CCK8 reagent contains a water-soluble yellow substance called formazan that can be reduced by dehydrogenase in the cellular mitochondria, which is proportional to the number of viable cells under certain conditions, so the cell viability can be determined by detecting the OD value of formazan at 450 nm. The results of CCK8 assay showed that overexpression of SHP2 could increase the OD values of MGC-803 cells and BGC-823 cells. To sum up, SHP2 in TAMs can enhance the proliferation of GC cells (Figure 2).

### SHP2 in TAMs could enhance the migration and invasion of GC cells

**Figure 3:**
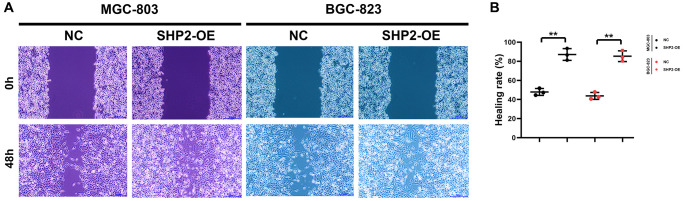
**SHP2 in TAM can promote the migration of gastric cancer.** (**A**) Results of wound healing assay at 0 h and 48 h; (**B**) Statistics of cell healing rate. ^**^P < 0.01.

**Figure 4:**
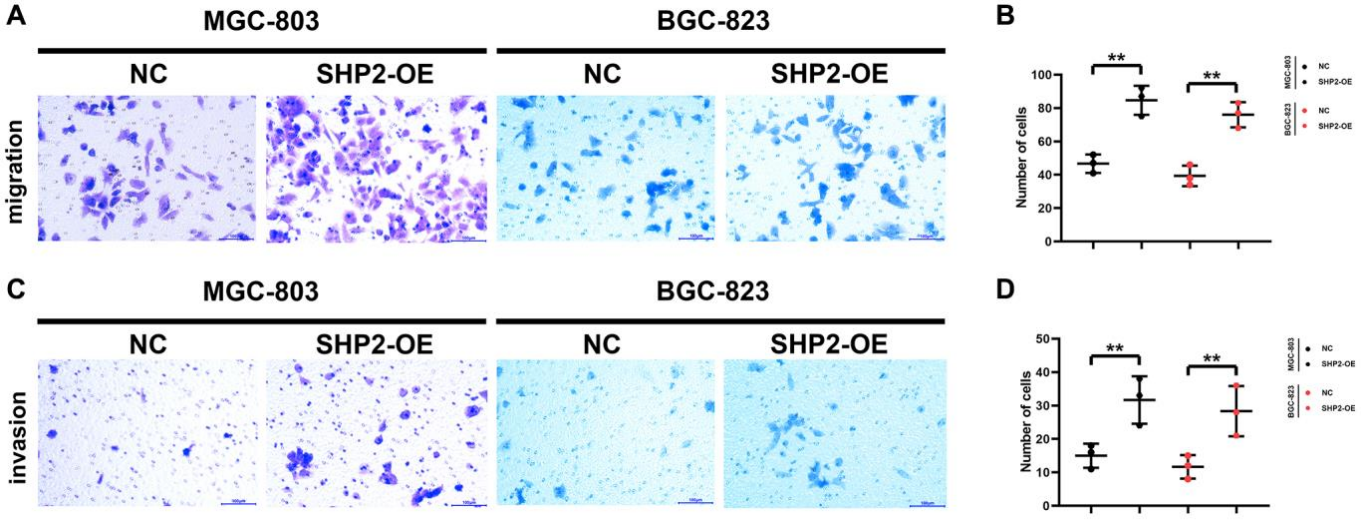
**SHP2 in TAM can promote the migration and invasion of gastric cancer.** (**A**) Results of the migration experiment; (**B**) Statistics of the number of migration cells; (**C**) Results of the invasion experiment; (**D**) Statistics of the number of invasion cells. ^**^P < 0.01.

A blank area called “scratch/wound” is artificially created on the monolayer cells, and the cells at the edge of the scratch/wound will gradually enter the blank area, so that the “scratch/wound” is healed. Images are captured at the beginning and during the cell migration process. The cell migration capacity is judged by measuring the scratch/wound distance at different time points. This process bears a similarity to the wound healing process *in vitro*, so it is also called wound healing assay, which can be utilized to observe the effects of exogenous factors such as drugs and genes on cell migration, repair and interaction. In this study, the results of wound healing assay revealed that overexpression of SHP2 could increase the wound healing rate of MGC-803 cells and BGC-823 cells (Figure 3). Besides, Transwell assay is to separate the high-nutrient culture medium from the low-nutrient culture medium using a layer of polycarbonate membrane. The cells are inoculated in the low-nutrient culture medium. Usually, the membrane pores are covered with Matrigel, which can mimic the ECM, and tumor cells must secrete hydrolase to pass through the filter membrane. The number of cells entering the lower chamber can reflect the migration and invasion capacity of tumor cells. In this study, the results showed that overexpression of SHP2 could increase the number of migrating and invading MGC-803 cells and BGC-823 cells (Figure 4). To sum up, SHP2 in TAMs can enhance the migration and invasion of GC cells.

### Mechanism of SHP2 in TAMs affecting the progression of GC

**Figure 5:**
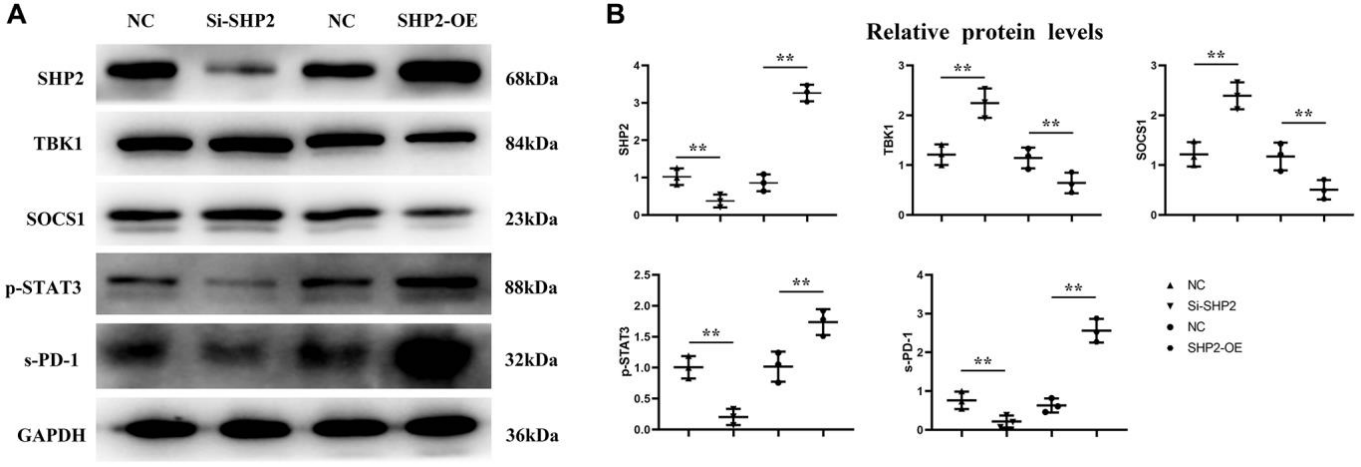
**Effect of SHP2 in TAM on p-STAT3, s-PD-1, TBK1 and SOCS1 expression.** (**A**) Western blotting results for SHP2, p-STAT3, s-PD-1, TBK1 and SOCS1; (**B**) Statistics of relative protein expression levels of SHP2, p-STAT3, s-PD-1, TBK1 and SOCS1. ^**^P < 0.01.

**Figure 6:**
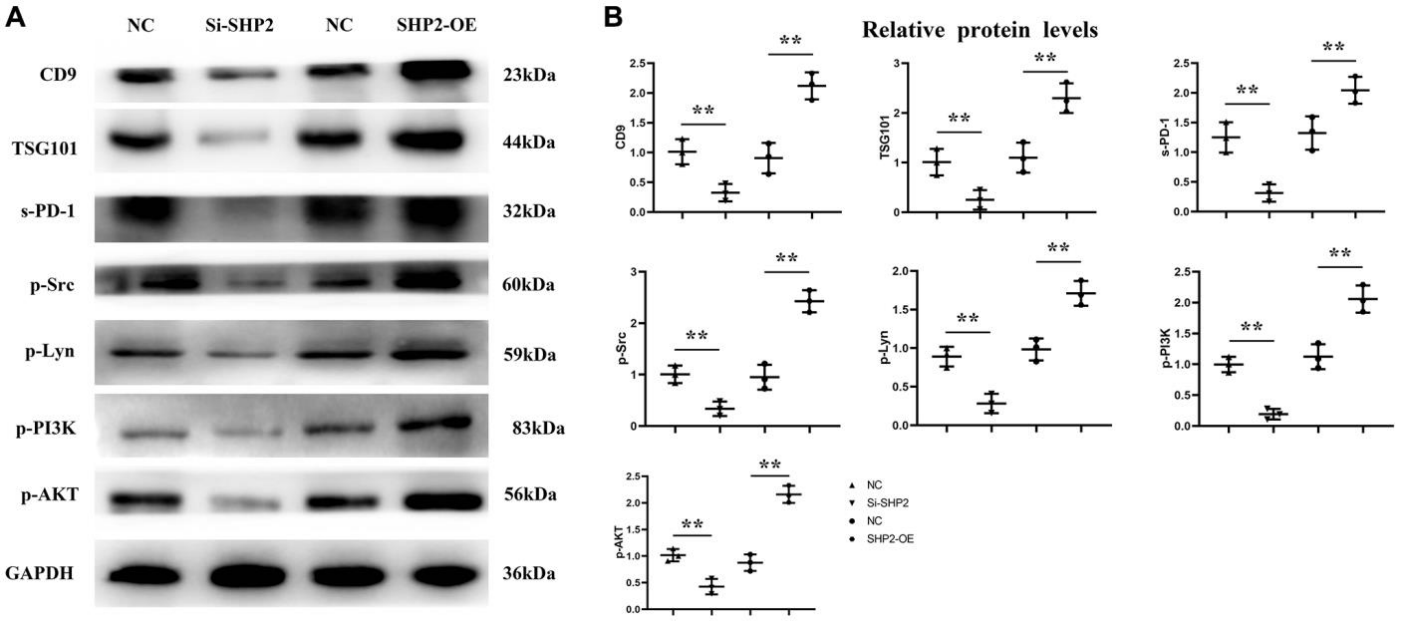
**Effect of SHP2 in TAM on the expression of CD9, TSG101, s-PD-1 in exosomes and the expression of p-Src, p-Lyn, p-PI3K and p-AKT in TAM.** (**A**) Western blotting results for CD9, TSG101, s-PD-1, p-Src, p-Lyn, p-PI3K and p-AKT; (**B**) Statistics of relative protein expression levels of CD9, TSG101, s-PD-1, p-Src, p-Lyn, p-PI3K and p-AKT. ^**^P < 0.01.

**Figure 7:**
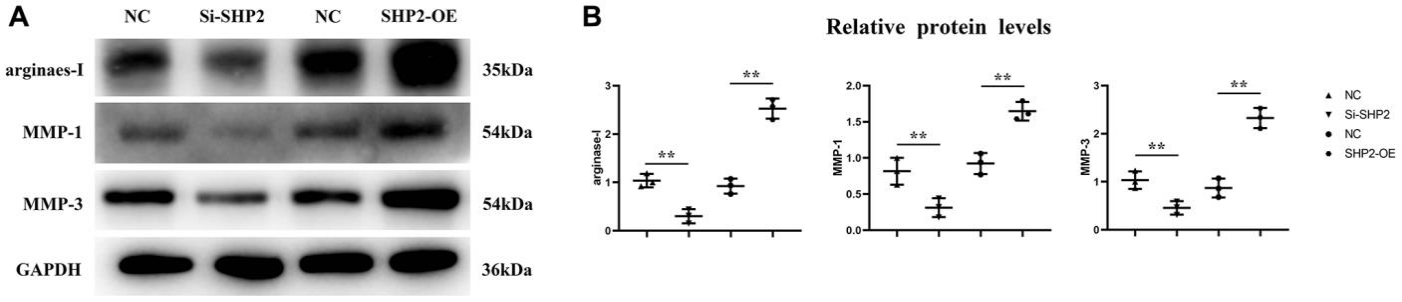
**Effect of SHP2 in TAM on the expression of Arginase-1, MMP1 and MMP3.** (**A**) Western blotting results for Arginase-1, MMP1 and MMP3; (**B**) The relative protein expression level of Arginase-1, MMP1 and MMP3. ^**^P < 0.01.

**Figure 8:**
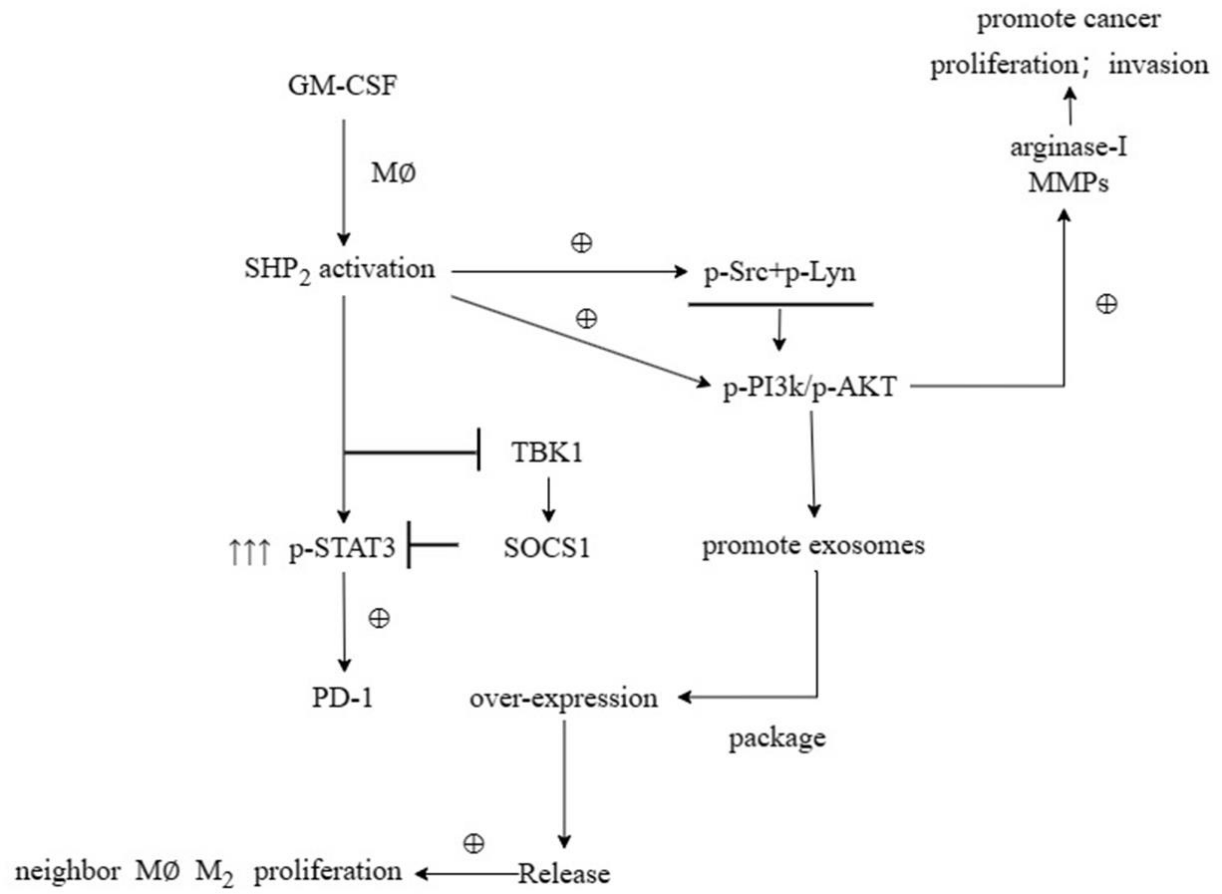
M-CSF secreted by gastric cancer cells promotes SHP2 in TAM to increase the secretion of exosomes, and also promotes the secretion of MMPs and Arginase-1 through the Src/PI3K/AKT axis, thereby promoting the M2 polarization and migration and invasion ability of TAM.

First, after the THP-1 cells were transfected with si-SHP2, SHP2-OE and NC, the exosomes were extracted and the expressions of related proteins were detected by Western blotting. It was found that compared with the NC group, the relative protein expression levels of SHP2, p-STAT-3 and s-PD-1 significantly decreased, while the relative protein expression levels of TBK-1 and SOCS-1 significantly increased in the si-SHP2 group. Compared with the NC group, the relative protein expression levels of SHP2, p-STAT3 and s-PD-1 significantly increased, while the relative protein expression levels of TBK1 and SOCS1 significantly decreased in the SHP2-OE group. It can be seen that SHP2 can inhibit TBK1 and SOCS1 and increase p-STAT3 and s-PD-1 (Figure 5). In exosomes, the relative protein expression levels of CD9, TSG101 and s-PD-1 significantly decreased in the si-SHP2 group but significantly increased in the SHP2-OE group compared with the NC group. In THP-1 cells, the relative protein expression levels of p-Src, p-Lyn, p-PI3K and p-AKT significantly decreased in the si-SHP2 group but significantly increased in the SHP2-OE group compared with the NC group (Figure 6). It can be inferred that SHP2 in TAMs can promote the secretion of exosomes and the M2 polarization of macrophages. In addition, in THP-1 cells, the relative protein expression levels of Arginase-1, MMP-1 and MMP-3 significantly decreased in the si-SHP2 group but increased in the SHP2-OE group compared with the NC group (Figure 7). It can be inferred that SHP2 in TAMs can facilitate the migration of GC cells by secreting MMPs through the PI3K/AKT signaling pathway. The above findings indicate that M-CSF secreted by GC cells promotes the secretion of SHP2 in TAMs to increase exosome secretion, and also promotes the secretion of MMPs and Arginase-1 through the Src/PI3K/AKT axis, thus facilitating the M2 polarization and migration and invasion capacity of TAMs (Figure 8).

## DISCUSSION

GC is one of the most common malignancies in the world, and also the second leading cause of cancer morbidity and mortality [15]. Despite an increasing incidence of gastroesophageal junction adenocarcinoma, gastric antral cancer remains the most common type of GC. The incidence of GC is gradually increasing in young people. GC patients exhibit characteristics of “three high and three low”, i.e., high incidence, metastasis and mortality rates, and low early diagnosis, radical resection and 5-year survival rates. The exact etiology of GC is unknown, and it is a fact that the majority of patients have been in the advanced stage [16], and some even lose the opportunity to undergo surgical resection. Advanced GC may also have a risk of metastasis, so the overall prognosis is unsatisfactory [17]. Many studies have been conducted in recent years to improve the prognosis of patients with GC, and seeking new molecules for targeted therapy is necessary.

Among a variety of adaptive and innate immune cells in the tumor microenvironment, macrophages are of particular importance due to their high abundance and negative roles in the progression of tumors. The M-CSF/CSF1R pathway is activated when M-CSF binds to the CSF1R expressed in myeloid cells. M-CSF in the tumor microenvironment promotes the M2 polarization of macrophages [18, 19]. Moreover, SHP1 and SHP2 are activated simultaneously in macrophages. In this study, the results of nude mouse tumor-bearing experiments showed that overexpression of SHP2 in TAMs could promote the progression of GC. Furthermore, the results of colony formation assay and CCK8 assay indicated that overexpression of SHP2 in TAMs could facilitate the proliferation of GC cells.

SHP099 enhances type I interferon signaling in macrophages by regulating the STING/TBK1/IRF3 pathway. Notably, the SHP2-mediated ubiquitin ligase TRIM27-induced degradation of TBK1 was investigated previously. Furthermore, it has been reported that SHP2 can directly bind to the kinase domain of TBK1 through the C-terminal domain (273e538, including the PTP domain), thereby inhibiting the phosphorylation of substrates. SOCS1 can promote the JAK2/STAT3 signaling pathway and enhance the expression levels of VEGFA, FGF2 and MMP9 in fibroblasts, and the activation of STAT3 can increase the expression of PD-1 [20, 21]. In this study, the results of Western blotting showed that in the si-SHP2 group, the relative protein expression levels of SHP2, p-STAT3 and s-PD-1 in TAMs were significantly lower, while the relative protein expression levels of TBK1 and SOCS1 were significantly higher than the NC group. In the SHP2-OE group, the relative protein expression levels of SHP2, p-STAT3 and s-PD-1 were significantly higher, while the relative protein expression levels of TBK1 and SOCS1 were significantly lower than the NC group. In exosomes, the relative protein expression levels of CD9, TSG101 and s-PD-1 were significantly lower in the si-SHP2 group than the NC group, while they were significantly higher in the SHP2-OE group than the NC group.

Phosphorylated SHP2 functions as a scaffold protein, recruiting Syk to Dectin-1 or Dectin-2/3 receptor complexes, which leads to the activation of Syk and Syk-dependent signaling pathways. SHP2 can also promote the activation of SRC. Src family kinases (SFKs) are the largest family of non-receptor kinases, which consists of nine members: Blk, Fgr, Fyn, Hck, Lck, Lyn, Src, Yes, and Yrk [22]. Activation of SRC can promote the phosphorylation of the PI3K/AKT signaling pathway, thus increasing the expression of MMPs. In this study, compared with the NC group, the relative protein expression levels of p-Src, p-Lyn, p-PI3K, p-AKT, Arginase-1, MMP1 and MMP3 significantly decreased in the si-SHP2 group but significantly increased in the SHP2-OE group. The above results were verified by both wound healing assay and Transwell assay. Zhao W and Liu Q mainly study GSK3 by downregulating TNFα-induced GM-CSF by downregulating ERK signaling, thereby driving tumor metastasis by regulating macrophage recruitment and activation. The axis with IL-34/M-CSF/M-CSFR is useful for regulating macrophage differentiation and thus influencing tumor progression [23, 24]. This study is different from these two papers, focusing on the effect of M-CSF secreted by GC cells on SHP2 and its related signaling pathways in TAMs in the tumor microenvironment.

In conclusion, M-CSF secreted by GC cells can increase the expression of SHP2 in TAMs, thereby promoting the secretion of exosomes and M2 polarization of TAMs as well as the proliferation, invasion, and migration capacity of GC cells.

## References

[1] Yusefi AR, Bagheri Lankarani K, Bastani P, Radinmanesh M, Kavosi Z. Risk Factors for Gastric Cancer: A Systematic Review. Asian Pac J Cancer Prev. 2018; 19:591–603. 10.22034/APJCP.2018.19.3.591 29579788 PMC5980829

[2] Zhang XY, Zhang PY. Gastric cancer: somatic genetics as a guide to therapy. J Med Genet. 2017; 54:305–12. 10.1136/jmedgenet-2016-104171 27609016

[3] Park JY, von Karsa L, Herrero R. Prevention strategies for gastric cancer: a global perspective. Clin Endosc. 2014; 47:478–89. 10.5946/ce.2014.47.6.478 25505712 PMC4260094

[4] Gao JP, Xu W, Liu WT, Yan M, Zhu ZG. Tumor heterogeneity of gastric cancer: From the perspective of tumor-initiating cell. World J Gastroenterol. 2018; 24:2567–81. 10.3748/wjg.v24.i24.2567 29962814 PMC6021770

[5] Kunisaki C, Akiyama H, Nomura M, Matsuda G, Otsuka Y, Ono HA, Takagawa R, Nagahori Y, Takahashi M, Kito F, Shimada H. Clinicopathological features of gastric carcinoma in younger and middle-aged patients: a comparative study. J Gastrointest Surg. 2006; 10:1023–32. 10.1016/j.gassur.2006.03.001 16843873

[6] Takatsu Y, Hiki N, Nunobe S, Ohashi M, Honda M, Yamaguchi T, Nakajima T, Sano T. Clinicopathological features of gastric cancer in young patients. Gastric Cancer. 2016; 19:472–8. 10.1007/s10120-015-0484-1 25752270

[7] Qian BZ, Pollard JW. Macrophage diversity enhances tumor progression and metastasis. Cell. 2010; 141:39–51. 10.1016/j.cell.2010.03.014 20371344 PMC4994190

[8] Xin L, Zhou LQ, Liu C, Zeng F, Yuan YW, Zhou Q, Li SH, Wu Y, Wang JL, Wu DZ, Lu H. Transfer of LncRNA CRNDE in TAM-derived exosomes is linked with cisplatin resistance in gastric cancer. EMBO Rep. 2021; 22:e52124. 10.15252/embr.202052124 34647680 PMC8647143

[9] Lin X, Wang S, Sun M, Zhang C, Wei C, Yang C, Dou R, Liu Q, Xiong B. miR-195-5p/NOTCH2-mediated EMT modulates IL-4 secretion in colorectal cancer to affect M2-like TAM polarization. J Hematol Oncol. 2019; 12:20. 10.1186/s13045-019-0708-7 30808369 PMC6390326

[10] Chan G, Kalaitzidis D, Neel BG. The tyrosine phosphatase Shp2 (PTPN11) in cancer. Cancer Metastasis Rev. 2008; 27:179–92. 10.1007/s10555-008-9126-y 18286234

[11] Idrees M, Oh SH, Muhammad T, El-Sheikh M, Song SH, Lee KL, Kong IK. Growth Factors, and Cytokines; Understanding the Role of Tyrosine Phosphatase SHP2 in Gametogenesis and Early Embryo Development. Cells. 2020; 9:1798. 10.3390/cells9081798 32751109 PMC7465981

[12] Ran H, Tsutsumi R, Araki T, Neel BG. Sticking It to Cancer with Molecular Glue for SHP2. Cancer Cell. 2016; 30:194–6. 10.1016/j.ccell.2016.07.010 27505669 PMC5558882

[13] Sun X, Zhang J, Wang Z, Ji W, Tian R, Zhang F, Niu R. Shp2 Plays a Critical Role in IL-6-Induced EMT in Breast Cancer Cells. Int J Mol Sci. 2017; 18:395. 10.3390/ijms18020395 28208810 PMC5343930

[14] Yu M, Xu C, Zhang H, Lun J, Wang L, Zhang G, Fang J. The tyrosine phosphatase SHP2 promotes proliferation and oxaliplatin resistance of colon cancer cells through AKT and ERK. Biochem Biophys Res Commun. 2021; 563:1–7. 10.1016/j.bbrc.2021.05.068 34052504

[15] Forman D, Burley VJ. Gastric cancer: global pattern of the disease and an overview of environmental risk factors. Best Pract Res Clin Gastroenterol. 2006; 20:633–49. 10.1016/j.bpg.2006.04.008 16997150

[16] Skierucha M, Milne AN, Offerhaus GJ, Polkowski WP, Maciejewski R, Sitarz R. Molecular alterations in gastric cancer with special reference to the early-onset subtype. World J Gastroenterol. 2016; 22:2460–74. 10.3748/wjg.v22.i8.2460 26937134 PMC4768192

[17] Carvalho R, Milne AN, van Rees BP, Caspers E, Cirnes L, Figueiredo C, Offerhaus GJ, Weterman MA. Early-onset gastric carcinomas display molecular characteristics distinct from gastric carcinomas occurring at a later age. J Pathol. 2004; 204:75–83. 10.1002/path.1602 15307140

[18] Cannarile MA, Weisser M, Jacob W, Jegg AM, Ries CH, Rüttinger D. Colony-stimulating factor 1 receptor (CSF1R) inhibitors in cancer therapy. J Immunother Cancer. 2017; 5:53. 10.1186/s40425-017-0257-y 28716061 PMC5514481

[19] Kulkarni A, Chandrasekar V, Natarajan SK, Ramesh A, Pandey P, Nirgud J, Bhatnagar H, Ashok D, Ajay AK, Sengupta S. A designer self-assembled supramolecule amplifies macrophage immune responses against aggressive cancer. Nat Biomed Eng. 2018; 2:589–99. 10.1038/s41551-018-0254-6 30956894 PMC6450396

[20] Shaulian E, Karin M. AP-1 as a regulator of cell life and death. Nat Cell Biol. 2002; 4:E131–6. 10.1038/ncb0502-e131 11988758

[21] Atsaves V, Leventaki V, Rassidakis GZ, Claret FX. AP-1 Transcription Factors as Regulators of Immune Responses in Cancer. Cancers (Basel). 2019; 11:1037. 10.3390/cancers11071037 31340499 PMC6678392

[22] Bard-Chapeau EA, Li S, Ding J, Zhang SS, Zhu HH, Princen F, Fang DD, Han T, Bailly-Maitre B, Poli V, Varki NM, Wang H, Feng GS. Ptpn11/Shp2 acts as a tumor suppressor in hepatocellular carcinogenesis. Cancer Cell. 2011; 19:629–39. 10.1016/j.ccr.2011.03.023 21575863 PMC3098128

[23] Liu Q, Zhang Y, Zhang J, Tao K, Hambly BD, Bao S. Inverse correlation between Interleukin-34 and gastric cancer, a potential biomarker for prognosis. Cell Biosci. 2020; 10:94. 10.1186/s13578-020-00454-8 32765828 PMC7399616

[24] Zhao W, Xiang Y, Zhang Z, Liu X, Jiang M, Jiang B, Song Y, Hu J. Pharmacological inhibition of GSK3 promotes TNFα-induced GM-CSF via up-regulation of ERK signaling in nasopharyngeal carcinoma (NPC). Int Immunopharmacol. 2020; 83:106447. 10.1016/j.intimp.2020.106447 32248019

